# Out of the Lab and into the Bathroom: Evening Short-Term Exposure to Conventional Light Suppresses Melatonin and Increases Alertness Perception

**DOI:** 10.3390/ijms14022573

**Published:** 2013-01-28

**Authors:** Amely Wahnschaffe, Sven Haedel, Andrea Rodenbeck, Claudia Stoll, Horst Rudolph, Ruslan Kozakov, Heinz Schoepp, Dieter Kunz

**Affiliations:** 1 Institute of Physiology, Charité–Universitätsmedizin Berlin (CBF), 10115 Berlin, Germany; E-Mails: sven@haedel.de (S.H.); andrea.rodenbeck@charite.de (A.R.); claudia.stoll@charite.de (C.S.); dieter.kunz@charite.de (D.K.); 2 Trilux GmbH & Co.KG, 59759 Arnsberg, Germany; E-Mail: hrudolph@trilux.de; 3 Leibniz Institute for Plasma Science and Technology (INP), 17489 Greifswald, Germany; E-Mails: kozakov@inp-greifswald.de (R.K.); Schoepp@inp-greifswald.de (H.S.); 4 German Heart Institute, 13353 Berlin, Germany

**Keywords:** melatonin, circadian rhythm, light, sleep disturbances, alertness

## Abstract

Life in 24-h society relies on the use of artificial light at night that might disrupt synchronization of the endogenous circadian timing system to the solar day. This could have a negative impact on sleep–wake patterns and psychiatric symptoms. The aim of the study was to investigate the influence of evening light emitted by domestic and work place lamps in a naturalistic setting on melatonin levels and alertness in humans. Healthy subjects (6 male, 3 female, 22–33 years) were exposed to constant dim light (<10 lx) for six evenings from 7:00 p.m. to midnight. On evenings 2 through 6, 1 h before habitual bedtime, they were also exposed to light emitted by 5 different conventional lamps for 30 min. Exposure to yellow light did not alter the increase of melatonin in saliva compared to dim light baseline during (38 ± 27 pg/mL *vs.* 39 ± 23 pg/mL) and after light exposure (39 ± 22 pg/mL *vs.* 44 ± 26 pg/mL). In contrast, lighting conditions including blue components reduced melatonin increase significantly both during (office daylight white: 25 ± 16 pg/mL, bathroom daylight white: 24 ± 10 pg/mL, Planon warm white: 26 ± 14 pg/mL, hall daylight white: 22 ± 14 pg/mL) and after light exposure (office daylight white: 25 ± 15 pg/mL, bathroom daylight white: 23 ± 9 pg/mL, Planon warm white: 24 ± 13 pg/mL, hall daylight white: 22 ± 26 pg/mL). Subjective alertness was significantly increased after exposure to three of the lighting conditions which included blue spectral components in their spectra. Evening exposure to conventional lamps in an everyday setting influences melatonin excretion and alertness perception within 30 min.

## 1. Introduction

Both nonorganic disturbances of the sleep–wake schedule (F51.2) and nonorganic insomnia (F51.0) are discrete psychiatric diagnoses according to ICD-10 chapter V, as well as common symptoms of various psychiatric disorders. Whereas the high incidence of the key feature—nonrestorative sleep—is a widely accepted fact [1], it was not until recently that the enormous socioeconomic costs related to sleep disorders were reported [2]. Recently approved melatonergic agents have proven efficient in patients suffering from major depression, as well as nonorganic insomnia, and thereby introduced the potential role of melatonin for psychiatric disorders into scientific discourse [3]. The hormone melatonin is central for the linkage between environmental and internal rhythms, because its secretion pattern depends on environmental light conditions, and an increase of its serum levels signals “night-mode” to numerous body functions. The question arises whether light exposure at the wrong time—e.g., artificial evening light—via suppression of melatonin may contribute to sleep-related psychiatric disturbances by suppressing melatonin secretion.

The earth’s rotation causes the most reliably recurrent event in nature: the daily light–dark cycle. The evolutionary result is a network of internal clocks governed by a master clock located in the hypothalamic suprachiasmatic nucleus (SCN), which drives the predictable part of daily physiological variations in a precise manner [4]. This circadian timing system (CTS) has been shown to be involved in the daily variation of almost every physiological and psychological system evaluated thus far (e.g., hormones, neurotransmitters, receptor densities and affinities, gene expression, mood, motor activity, pharmacokinetics, pharmacodynamics, responses to pharmacological treatment) [5–10].

Maintaining synchronized circadian rhythms is important to health and well-being. A growing body of evidence suggests that desynchronization of circadian rhythms may play a role in various tumoral diseases, diabetes, obesity, depression, and Alzheimer’s disease [11–14]. Shift workers, who serve as a model for internal desynchronization, are subject to increased morbidity and mortality from a number of diseases, including cardiovascular disorders and cancer [15–17].

The daily light–dark cycle synchronizes the CTS with the natural 24-h day [18]. In 2000, melanopsin was identified in a subgroup of retinal ganglion cells, which were originally only known to integrate the information from rods and cones and transform it into neuronal activation [19]. This allowed for a non-image-forming system to be characterized in accretive detail: light and darkness are predominantly perceived by specialized intrinsic photosensitive retinal ganglion cells expressing melanopsin. On the one hand, they react directly to light input; on the other hand, they gather light information from rods and cones. This integrated measurement provides the internal clock with information on the time and length of day [20]. The blue portion of the visible spectrum appears to play the dominant role in the intrinsic photosensitivity of these cells, as well as for the integrated non-image-forming response to light stimuli [21]. Two groups independently reported an action spectrum for light-induced melatonin suppression by monochromatic light at night [22,23]. Additionally, monochromatic blue light has been shown to induce pupillary constriction, increase heart rate, influence thermoregulation, enhance alertness, change the frequency of the electroencephalogram and influence sleep architecture [24–26]. In contrast to green light, blue light was even shown to increase human *PER2* gene expression after 2 h of evening light exposure [27]. Enezi *et al*. recently suggested a “melanopic” spectral efficiency function as an alternative to lux and photon density for describing the impact of polychromatic light of different spectral distributions on melanopsin expression and the proximate pupillomotor and circadian responses [28]. This melanopic function was successfully applied to rod- and coneless mice, but not yet to wild-type mice or even humans. Effects of long-term polychromatic light on human melatonin levels have already been shown: a study using artificial room light demonstrated that light exposure <200 lux for 8 h until bedtime delayed the melatonin onset without shifting melatonin offset [29]. Shanti *et al.* [30] reported a wavelength-dependent influence of 4 h of evening light exposure on melatonin suppression, subjective sleepiness, and sleep onset latency. However, data on short-term evening polychromatic light exposure are still missing.

Many practical clinical trials have shown the effects of light as a therapeutic agent on depression, age-related sleep problems and agitation in dementia (see, for example, [31–34]). Thus, evening light exposure was successfully applied to increase alertness in older adults with evening sleepiness [35]. Nevertheless, most of the studies in the context of light influences on circadian rhythms were performed using long-term, high intensity bright white, polychromatic or blue light in artificial settings in a way that seldom occurs even in our world of artificial lighting [22,23,36]. Even when the design for these studies was modeled on average exposition under natural conditions for the periods they examined [30] they did not consider the impact of very short periods of light exposure on circadian parameters. Yet the evening setting can include relevant changes in lighting conditions such as a transition from a brightly lit gym to a moderately lit home, or from a dimly-lit reading corner or television light in the living room to the neon-lit bathroom, where you get ready for bed. Their findings are therefore difficult to generalize to such conditions. Other studies addressed the therapeutic potential of strengthening circadian rhythms with light. The question could be raised if simple behavior recommendations regarding “light hygiene” could already work as a therapeutic agent on circadian rhythm associated disturbances.

The hypotheses of the present study are that (1) light emitted by conventional home and work place lamps in a naturalistic evening setting can suppress melatonin excretion in healthy human subjects after very short periods of time; and (2) the strength of this influence is wavelength and intensity-dependent and can be minimized by blue depletion and intensity reduction. In a second step, we conduct an exploratory investigation of different lamps on subjective calmness, contentedness and alertness.

## 2. Results and Discussion

### 2.1. Raw Data and Descriptive Statistics

**Figure 1:**
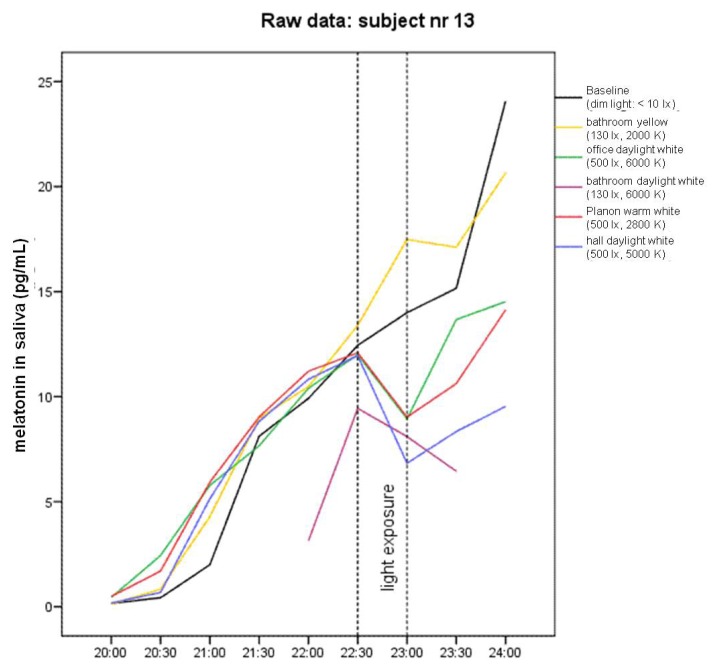
Raw data of melatonin levels in saliva in subject number 13 (subjects in the present study were numbered 7–15 following a pilot study with subjects 1–6). Lines represent the different lighting conditions.

Graphical analysis of melatonin raw data in most of the nine single subjects and in group means shows differences between baseline (dim light) and yellow bathroom lamp, on the one hand, and between baseline and the four lamps with blue components of the visible spectrum, on the other hand (e.g., Figure 1, subject 13).

**Figure 2:**
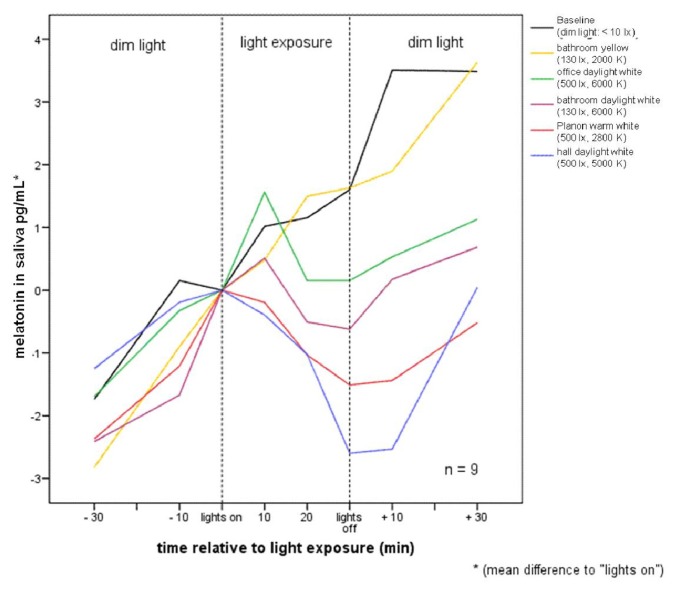
Group means of saliva melatonin concentrations; lines represent the different lighting conditions. Data are given relative to the zero point that represents melatonin concentrations at “lights on”. Values on *X*-axis represent mean differences of melatonin concentrations to “lights on”.

Mean group changes of melatonin levels relative to “lights on” are given in Figure 2. While melatonin concentrations continuously rise during the time of light exposure under baseline and yellow bathroom lamp conditions, the increase of melatonin concentrations stops or is even reversed during exposure to any of the four lamps, including blue portions, starting already as early as 20 min after “lights on”. After termination of light exposure, melatonin concentrations start rising again, but not to the level reached within 30 min after baseline or yellow bathroom lamp concentrations, which are within 30 min after lights exposure. Nearly the same changes between lighting regimes are reached by using the mean between the melatonin concentrations 10 min before “lights on” and “lights on” as zero point (see means and standard deviations in Table 1). This applies both to the analysis of all subjects and to the analysis including the six subjects with complete data for all lighting conditions. The mean difference of melatonin concentration between 10 min before “lights on” and “lights on” was 0.68 ± 0.47 pg/mL (range: −0.12 to 1.34 pg/mL per subject).

### 2.2. Data Analysis

#### 2.2.1. Melatonin Levels in Different Lighting Regimes

**Figure 3:**
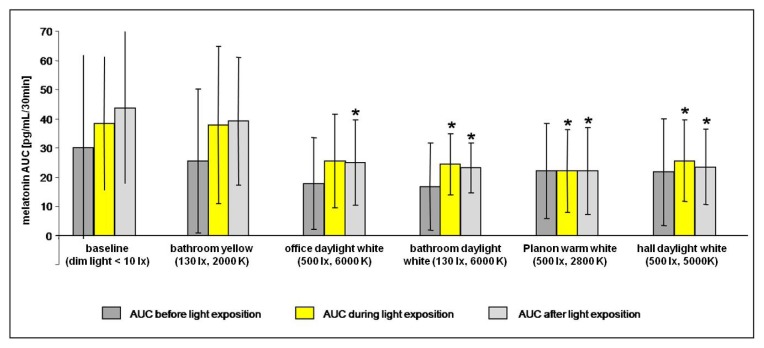
Melatonin levels before, during, and after light exposure. Areas under curve (AUC) of melatonin saliva levels before (AUC pre, 30–0 min before lights on), during (AUC light, 0–30 min after lights on), and after (AUC post, 0–30 min after lights off) the light exposure. Data are expressed as means ± standard deviation; *p < 0.05 compared to baseline, *n* = 6.

Friedman ANOVA was only applied to six of the nine subjects, because the melatonin in saliva data sets of three subjects were incomplete due to an insufficient amount of saliva in the assays. In the six subjects with complete melatonin data sets, Friedman ANOVA revealed significant group differences in melatonin levels during (χ^2^ = 15.5238, *p* < 0.009) and after (χ^2^ = 18.09524, *p* < 0.003) light exposure. In contrast, groups did not differ prior to light exposure with respect to the different lighting regimes (χ^2^ = 9.04719, *p* < 0.11), indicating comparable baselines each evening (see Figure 3). Thus, an order effect induced by the experiment can be ruled out. Two out of three Friedman ANOVAs showed significant differences between lighting conditions, thereby indicating a high overall significance (*p* = 0.0072) of the comparisons.

Regarding the time during light exposure, post-hoc comparisons indicate significant decreases of melatonin levels during lighting with three of the four lamps including blue components (daylight white hall, daylight white bathroom, and warm white Planon) as compared to baseline (*p* < 0.03 each). The decrease during exposure to daylight white office failed to reach significance (*p* = 0.07), while the yellow bathroom showed no effect on melatonin levels. *Post–hoc* analysis of the time after “lights off” showed significant (*p* < 0.03 each) lower melatonin levels for daylight white hall, daylight white bathroom, and warm white Planon as compared to baseline. Furthermore, AUC of melatonin levels was diminished following daylight white office (*p* < 0.05), while no changes following yellow bathroom could be detected (Figure 3).

#### 2.2.2. Subjective Alertness, Calmness and Contentedness in Different Lighting Regimes

**Figure 4:**
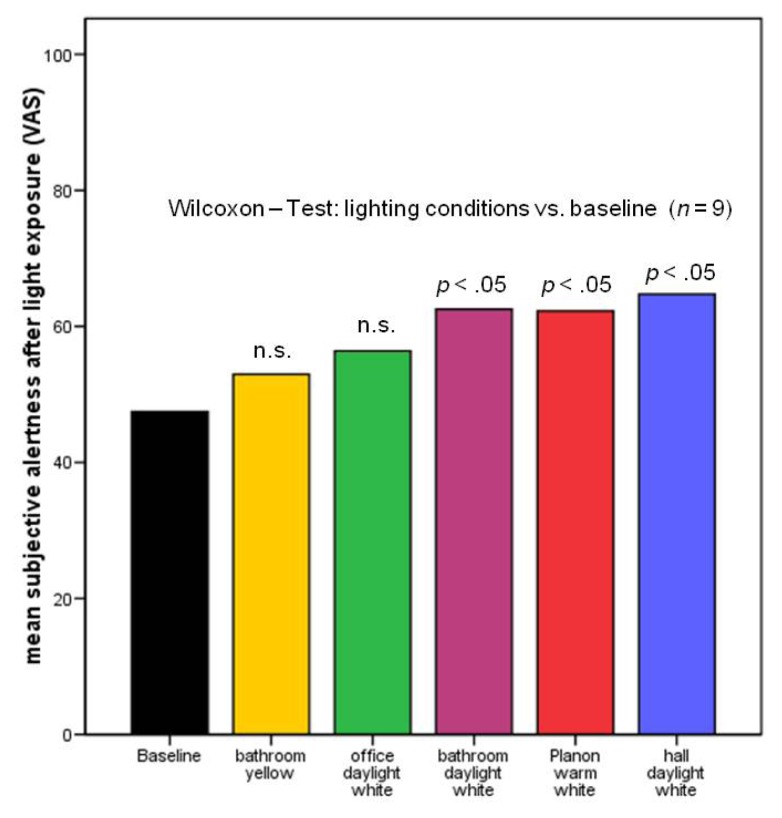
Subjective alertness after light exposure. Subjective alertness was measured by visual analog scales [37]. Data are expressed as means ± standard deviation; * *p* < 0.05 compared to baseline (Wilcoxon signed ranks-Test), *n* = 9.

The exploratory analysis of subjective calmness and contentedness did not yield any significant results. The results regarding subjective alertness are given in Figure 4. There were no significant differences between the baseline condition and the yellow bathroom condition, nor between baseline and daylight white office. In contrast, subjective alertness after light exposure was significantly increased compared to the baseline condition in three of the four lighting conditions that included a blue portion of the visible spectrum.

### 2.3. Discussion

The results of the present study show that short-term polychromatic light emitted by conventional lamps in the evening in a naturalistic setting can suppress melatonin secretion and increase subjective alertness. It is noteworthy, however, that subjects in our study were exposed to only 30 min of light in addition to dim light from 7:00 p.m. until midnight. On a descriptive level, these differences begin to appear after only 20 min of light exposure (see Figure 2).

**Figure 5:**
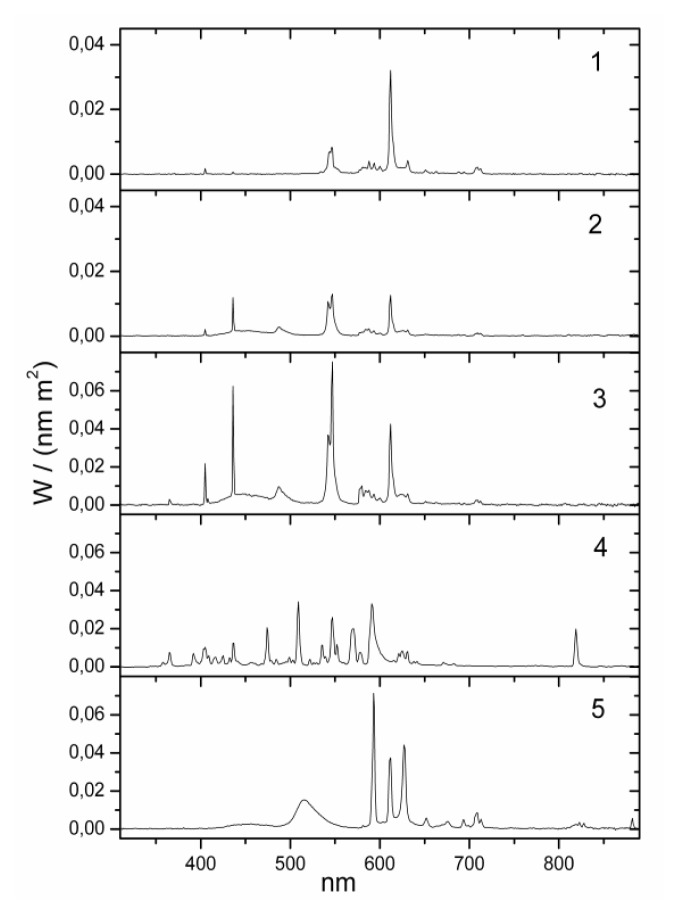
Spectral distributions. Numbers represent the lamps as follows: 1. bathroom yellow; 2. bathroom daylight white; 3. office daylight white; 4. hall daylight white; 5. Planon warm white.

The explanatory power of the presented data is limited by the small sample size, the non-systematic selection of lamps, and the merely subjective measurement of behavioral variables. To draw substantiated conclusions on adverse effects of light regarding health conditions, more lamps with a systematic variation of light intensity and spectral distribution reflecting the whole range of naturalistic evening light conditions should be tested on a bigger sample and with additional outcome variables such as validated vigilance tasks, polysomnographic sleep parameters, or cortisol levels. In our present sample, the influence of a lamp with no blue component and low light intensity (130 lx) on melatonin concentrations and on subjective alertness was close to zero (see yellow bathroom lamp in Table 2 and Figure 5), while melatonin secretion was suppressed by light with equal low intensity but a high blue component (white bathroom, 130 lx, 6000 K). In addition, all lamps with high (500 lx) light intensities had a detectable influence on melatonin concentrations after light exposure. Even the lamp with the lowest blue proportion (warm white Planon, 2000 K) of the high-intensity (500 lx) lights had strong effects on both melatonin secretion and subjective alertness. The rather low blue component of the warm white Planon—at this intensity (see Table 3 and Figure 5) and the relatively close distance of subjects’ eyes to the lamp (1 meter)—seems to be sufficient to cause this effect. The lamp with high blue component and high intensity (hall daylight white) produces, as expected, even more pronounced effects. In spite of the high light intensity and high blue component of the daylight white office lamp, it only reduced melatonin concentrations significantly after, but not during light exposure, and the increase of subjective alertness was not significant. The values of irradiance or melanopic lux (see Table 3), a measure that integrates light intensity and spectral distribution in one unit on an empirical basis [28], would imply a different ranking of the lamps than the amount of their effects in our sample. Thus, the expected combined influence of light intensity and spectral distribution [37] is not reflected in detail by our sample. This is probably due to the small sample size, combined with the pronounced individual differences [30].

Still, in none of the conditions exposing subjects to light containing a high proportion of blue or bright light was the rise in melatonin levels as large as they were in conditions exposing only to dim or yellow light.

Nevertheless, subjects varied substantially in the magnitude of melatonin suppression induced by any one lighting condition. Because all subjects were studied during the same week and exposed to their assigned lighting condition each evening during the experimental period, light history would only differ slightly between subjects according to their daytime activities [38]. Chemical agents or age-related changes in the lens of the eye can be ruled out [39,40]. It might therefore be argued that the observed differential responses to light have predominantly been due to individual variations in light sensitivity [41,42]. Experimental data in extreme chronotypes have shown that circadian rhythms differ in their response to chemical and environmental phase-shifting agents at the cellular level [43]. Though only suppression of melatonin as a circadian signal was investigated and not phase shifts of melatonin, it would be interesting to validate the diagnostic properties of the setting and paradigm used in the present study. This might help detect circadian rhythm sleep disorders by determining patients’ sensitivity to melatonin suppression by light. Regarding future research, it also seems worthwhile to think about potential long-term consequences. Though still not generally accepted, several studies suggest that melatonin in humans plays a similarly pivotal role to that already demonstrated in animals [44,45]. Low melatonin is associated with numerous diseases [46], such as cancer [17], and melatonin concentrations are reduced in the elderly [47], in patients with chronic primary insomnia [48–50], and even more so in patients suffering from Alzheimer’s dementia [51,52]. Even the neuroprotective properties of melatonin receive increasing evidence [53]. It is also well established that the increase in melatonin levels that occurs in the evening facilitates sleep induction [54–56]. Thus, it is tempting to speculate that melatonin suppression by artificial light during the evening plays a role in the sleep-related problems from which millions of individuals currently suffer. Nevertheless, the general causal relations of light, melatonin-levels, and the health conditions mentioned above still need to be determined.

The current study demonstrates that light affects not only melatonin levels, but also subjective alertness. However, this is thought to rely on a different pathway than the retinal–pineal pathway. As an fMRI study suggests, light directly activates cortical networks that underlie alertness [57]. This relationship requires further investigation with objective measurements.

Diurnal species such as humans are obliged to use their eyes to find their way about the world. Unlike nocturnal species like rats or bats, humans are at the mercy of their enemies when moving about during the nighttime hours. It is most likely for this reason that the CTS promotes motor inactivity and sleep during this period [4]. Although artificial light at night is clearly of great benefit to society, its inappropriate timing might alter human physiology. In December 2007, the World Health Organization added overnight shift work to its list of probable carcinogens [58]. One possible, but yet to be proven, cause of cancer in overnight shift workers is the reduction of nighttime melatonin resulting from exposure to artificial light. The results of the present study raise a question that should be addressed in detail by future research: do we all suffer from these effects to some degree due to nighttime exposure to blue and/or bright light?

## 3. Experimental Section

The study was performed in the sleep laboratory of the Institute of Physiology at the Charité in the Sankt Hedwig Hospital, Berlin, in February of 2007. The protocol was approved by the local ethics committee. Experimental procedures were conducted in accordance with the Declaration of Helsinki, and all participants gave their written, informed consent.

A total of 9 healthy subjects (3 women, 6 men, aged 22–33 years, mean = 26.3, SD = 4.2) participated in the study. Subjects were recruited by word-of-mouth recommendation and received pecuniary compensation. Medical, psychiatric, and sleep assessments were performed, including evaluations of sleep quality (Pittsburgh Sleep Quality Index, or PSQI [59]), of chronotype (Horne and Östberg [60]), and of seasonality (Seasonal Pattern Assessment Questionnaire, or SPAQ [61]). The inclusion criterion was a habitual bedtime between 10:00 p.m. and 1:00 a.m. (PSQI: Item 1—“When did you habitually go to sleep during the last four weeks?”). Exclusion criteria were age <18 or >35; current pregnancy; current or recent shift work (during the last year); poor sleep hygiene (sleep log or actigraphic proof of a more than 1-h deviation from habitual bedtime during a 7-day entrainment period); definite morning or evening types; seasonality score greater than 5; transmeridian travel (during, or within 1 month of, the study); any psychiatric, medical, or sleep disorder, including drug or alcohol abuse; and the intake of any medication, including over-the-counter medication, over the past month.

During a 7-day entrainment period, subjects were asked to keep a regular sleep–wake schedule with a tolerance range of ± 60 min of their self-estimated habitual bedtime. Compliance was monitored using a sleep log and actimetry (Actiwatch, Cambridge Neurotechnology).

During the 6-day experimental phase, we tried to create conditions that were as natural as possible: subjects followed their habitual daytime schedules, only attending the laboratory in the evening hours from 7 p.m. until midnight (monitored by sleep log and actimetry). Subjects were allowed to eat until 7 p.m. and to drink caffeinated beverages until 3 p.m. Alcoholic beverages were forbidden during lab days. Drinking water was accepted until 10 min prior to saliva collection. Subjects spent their time in the laboratory pursuing self-chosen activities (e.g., playing games, talking, watching videos on a dim-light screen) in a dimly lit room (<10 lx) by ordinary dimmable halogen lamps. Because circadian phase is associated with habitual bedtime, light exposure was timed individually to occur 1 h before habitual bedtime [62].

During evening 1, subjects were exposed only to dim light (baseline condition). During evenings 2 through 6, everyday lamps of different types (office, bathroom, industry), with two different intensities (130 *vs.* 500 lx at the cornea) and spectral distributions (4 with various and 1 without blue portions) were used to expose subjects, in randomly assigned groups of 3, to each lighting condition for a total of 30 min (for study design, see Table 2; for a detailed description of the lamps, see Table 3 and for their spectral distributions, see Figure 5). Three of the lamps were selected by their original application as examples to reflect naturalistic lighting conditions as occurring in bathrooms (bathroom daylight white), office work places (office daylight white) and industrial work places or gyms (hall daylight white). Two of the lamps were custom-made to produce: (1) a zero blue component bathroom light situation (bathroom yellow); and (2) a low blue component but high light intensity work place light situation (Planon warm white).

During light exposure, subjects were sitting at an assigned spot and were instructed to keep their gaze straight (at approximately 90°) over the 30-min light exposure. No other activity was permitted except talking. Subjects were informed that bright light may suppress melatonin; no information was given regarding the effects of light spectrum. Lighting conditions were characterized using a luxmeter (LMT Berlin), a spectrometer (StellarNet EPP-2000), and a luminance camera (LMK Mobile Advanced, Ilmenau). Light intensity and spectral distribution were determined for each lamp at the site of the experiment from the position of subjects’ eyes, *i.e.*, in the direction and angle of the gaze (performed by INP, Greifswald, Germany).

Each evening during the study period, self-assessment of alertness, calmness and contentedness was performed every 30 min using a paper and pencil version of visual analogue scales [63]. The VAS assessment at the start of light exposure was brought forward by 5 min to avoid disturbing the exposure procedure. In addition, saliva was collected with salivettes every 30 min during dim-light exposure, as well as 10 min before, every 10 min during, and 10 min after light exposure. Saliva collection never took more than one minute. Melatonin concentration in saliva was determined by a commercial radioimmunoassay (Bühlmann Laboratories, Allschwill, Switzerland) with an analytical least detectable dose of 0.15 pg/mL, intraassay precision: 6.7% (range: 4.9%–8.8%), interassay precision: 10.4% (range: 8.5%–13.9%).

As a consequence of the design, subjects’ bedtimes were slightly delayed during the experiment, as they still needed to get home after midnight. We tried to rule out possible order effects by using a crossover design and by also controlling for differences between lighting conditions and light exposition (see results section for details).

### Statistical Analysis

In order to calculate descriptive group differences, the reference point for differential melatonin levels was set to zero for each individual: (1) at time of “lights on” (see Figure 2); and (2) at the mean of 10 min before “lights on” and “lights on” (see Table 1). For statistical analysis, melatonin concentrations were calculated as areas under the curve (AUC) before, during, and after light exposure. AUC before light exposure (AUC pre) included 30 to 0 min before “lights on,” based on 20- and 10-min intervals. AUC during lighting (AUC light) included 10 to 30 min after “lights on,” based on 10-min intervals. AUC after light exposure (AUC post) included 0 to 30 min after “lights off,” based on 10- and 20-min intervals. Friedman ANOVAs were calculated for each AUC (pre, light, and post), followed by *post–hoc* Wilcoxon-tests. Friedman ANOVAs were alpha-adjusted using the method of Cross and Chaffin [64]. Three subjects had to be excluded from statistical analysis of melatonin levels due to single missing data caused by incorrect saliva collection.

For explorative purposes, subjective alertness was compared between baseline and the various lighting conditions using Wilcoxon signed-rank test.

## 4. Conclusions

Low-intensity light emitted by everyday lamps in a naturalistic setting can influence melatonin levels and alertness perception in healthy human subjects after only 30 min. The suppressive effect of this light exposure on melatonin can be minimized by using a yellow lamp without blue component. The order of the other lamps in the degree of influence on alertness and melatonin concentrations in our sample is not completely as expected, which could be due to the small sample size. It should be replicated with bigger sample sizes, also including further outcome variables such as sleep parameters and objective measures of behavioral variables. This could highlight the impact of evening light on adverse health outcomes in more depth. Still, even with this small sample size, the melatonin suppressing and alertness enhancing power of short exposition to ordinary lamps in contrast to dim light or yellow light without blue component could be shown in this study. We suggest that potentially adverse biological effects of untimely light exposure could be reduced or avoided by appropriate tuning of the spectral content and intensity of lighting devices, similar to the way lamps with specially designed spectral distributions can be used therapeutically in order to relieve various conditions. To put it—albeit tentatively—in a nutshell: filter out blue or dim the light when you go brush your teeth at night!

## Figures and Tables

**Table 1:** Group means and standard deviations of saliva melatonin concentrations. Data are given relative to the zero point representing the mean of melatonin concentrations in saliva 10 min before and at “lights on”. The first part of the table gives values for the entire sample, and the second part only for the six subjects with complete data sets.

		Pre −60	Pre −30	Mean −10 to 0 min before lights on	10	20	30	Post +10	Post +30
Part 1	Baseline (*n* = 9)	(−4.21) ± 1.89	(−2.26) ± 1.67	0	0.94 ± 2.47	1.08 ± 2.20	1.525 ± 1.69	3.48 ± 2.34	3.41 ± 1.78
	bathroom yellow (*n* = 9)	(−4.40) ± 2.68	(−2.37) ± 2.04	0	0.93 ± 1.56	1.95 ± 0.64	2.08 ± 1.15	2.075 ± 2.96	4.085 ± 4.26
	office daylight white (*n* = 8)	(−3.33) ± 2.23	(−1.29) ± 1.88	0	1.97 ± 2.90	0.56 ± 1.55	0.56 ± 2.48	0.94 ± 1.57	1.54 ± 2.23
	bathroom daylight white (*n* = 8)	(−4.05) ± 1.99	(−2.03) ± 1.44	0	0.97 ± 1.15	(−0.12) ± 1.00	(−0.13) ± 1.72	0.62 ± 2.96	2.03 ± 4.28
	Planon warm white (*n* = 9)	(−3.68) ± 2.94	(−1.77) ± 1.98	0	0.41 ± 2.12	(−0.43) ± 1.72	(−0.91) ± 1.64	(−0.83) ± 2.07	0.09 ± 2.83
	hall daylight white (*n* = 8)	(−2.66) ± 1.12	(−1.15) ± 0.76	0	(−0.30) ± 1.30	(−1.475) ± 1.42	(−2.50) ± 2.11	(−3.23) ± 4.675	(−0.98) ± 6.09

Part 2	*n* = 6								
	Baseline	(−3.40) ± 1.54	(−1.88) ± 1.06	0	1.58 ± 1.87	1.42 ± 1.44	1.66 ± 1.92	3.84 ± 2.48	2.67 ± 1.58
	bathroom yellow	(−5.18) ± 2.91	(−2.76) ± 2.33	0	0.55 ± 1.80	2.02 ± 0.74	2.29 ± 1.39	1.18 ± 3.30	2.38 ± 3.72
	office daylight white	(−2.96) ± 2.39	(−0.97) ± 2.11	0	2.45 ± 3.24	0.37 ± 1.78	0.61 ± 2.94	0.885 ± 1.85	1.24 ± 2.14
	bathroom daylight white	(−4.295) ± 2.07	(−2.16) ± 1.65	0	0.58 ± 0.85	(−0.11) ± 1.01	(−0.505) ± 1.86	(−0.535) ± 1.79	0.22 ± 2.47
	Planon warm white	(−2.95) ± 1.43	(−0.97) ± 1.31	0	0.98 ± 0.93	0.02 ± 0.69	(−0.89) ± 1.45	(−0.68) ± 1.17	0.27 ± 1.39
	hall daylight white	(−2.31) ± 1.06	(−1.02) ± 0.83	0	(−0.26) ± 1.53	(−1.29) ± 1.58	(−2.09) ± 2.09	(−1.69) ± 2.05	1.04 ± 3.60

**Table 2:** Study design.

Group	Day 1	Day 2	Day 3	Day 4	Day 5	Day 6
I	Baseline:	hall lighting	office lighting	bathroom	bathroom	Planon
II		office lighting	bathroom	bathroom	Planon	hall lighting
II		bathroom	bathroom	Planon	hall lighting	office lighting

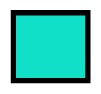
 daylight white 
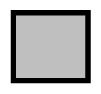
 warm white 
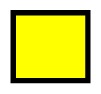
 yellow

**Table 3:** Lighting conditions.

No.	lamp name	Lux (lx)	Kelvin (K)	Irradiance (W/m^2)^	Lux_m_ (lx_m_) 1	Type	Model	Blue component	Position	Eye (cm) distance 2	Company
0	baseline (dim light)	<10						normal	pointing to ceiling	150 cm	
1	bathroom yellow	130	2000	0.319	118	fluorescent	Custom-made	zero	wall over mirror	100 cm	Narva BEL
2	bathroom daylight white	130	6000	0.385	581	fluorescent	6641 L18/W	high	wall over mirror	100 cm	Narva BEL
3	office daylight white	500	6000	1.437	2069	fluorescent (3 pieces)	T5 54W/860	high	ceiling	150 cm	Trilux Narva BEL
4	hall daylight white	500	5000	1.391	1724	metal halogenid	NCT 70W	high	ceiling	150 cm	Trilux Narva GLE
5	Planon warm white 3	500	2800	1.432	1826	dielectric inhibited	custom made	low	wall	100 cm	Osram

1 The unit melanopic lux (lx_m_) was recently suggested by al Enezi *et al.* to predict the sensitivity of melanopsin photoreceptors to polychromatic light [28]. It was derived from action spectra of monochromatic lights in several species;

2 Due to reflection, the whole visual field was exposed with a maximum in the center;

3 The Planon is an advanced prototype dielectric inhibited light source. It is being developed by Osram, Munich. So far, more than 150 lamps have been built. It is currently being used as a rugged light source in CNC Machines.
